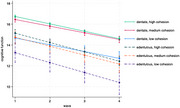# Social context matters: Neighborhood environment as a moderator of the longitudinal relationship between edentulism and cognitive function among older adults in the United States

**DOI:** 10.1002/alz70858_101278

**Published:** 2025-12-25

**Authors:** Ruotong Mona Liu, Xiang Qi, Weiyu Mao, Huabin Luo, Sophie Zhijing Xu, Bei Wu

**Affiliations:** ^1^ New York University, New York, NY, USA; ^2^ University of Nevada, Reno, Reno, NV, USA; ^3^ East Carolina University, Greenville, NC, USA

## Abstract

**Background:**

Previous research has shown poor oral health and neighborhood environment are both risk factors for cognitive decline among older adults. Little research has assessed the synergistic effects of poor oral health and neighborhood environment on cognitive health. This study examined whether neighborhood environment moderates the relationship between edentulism and cognitive function over time.

**Method:**

Using data from the Health and Retirement Study, we analyzed 9,929 adults aged 60 and older with 39,976 person‐wave observations over 14 years (2006‐2020). Cognitive function was measured using the modified Telephone Interview for Cognitive Status. Edentulism status was self‐reported complete tooth loss. Neighborhood environment included perceived neighborhood cohesion and disorder. We used linear mixed‐effect models to examine the moderation effect of neighborhood environment on the association between edentulism and cognitive function over time.

**Result:**

Edentulous participants (22.4%) showed an accelerated decline over time in cognitive function compared to dentate participants (β=‐0.57, 95% CI: ‐0.98, ‐0.16). The analysis revealed that neighborhood cohesion moderated the relationship between edentulism and cognitive function over time (β=0.08, 95% CI: 0.01, 0.15). Specifically, among individuals reporting higher neighborhood cohesion, the negative effect of edentulism on cognitive decline was attenuated. Neighborhood disorder had no significant associations with cognitive function.

**Conclusion:**

This study demonstrates the relationship between edentulism and cognitive function varies by levels of neighborhood cohesion. The findings highlight the significance of neighborhood context in understanding the relationship between oral health and cognitive aging and suggest interventions addressing community environment may be particularly relevant for older adults with oral health challenges.